# Barriers to Trace-back in a Salad-associated EHEC Outbreak, Sweden, June 2013

**DOI:** 10.1371/currents.outbreaks.80bbab3af3232be0372ea0e904dcd1fe

**Published:** 2014-06-06

**Authors:** Michael Edelstein, Camilla Sundborger, Maria-Pia Hergens, Sofie Ivarsson, Rikard Dryselius, Mona Insulander, Cecilia Jernberg, Yvan Hutin, Anders Wallensten

**Affiliations:** Public Health Agency of Sweden and European Programme for Intervention Epidemiology Training (EPIET), European Centre for Disease Prevention and Control (ECDC), Stockholm, Sweden; Public Health Agency of Sweden, Stockholm, Sweden; Department of Communicable Disease Control, Stockholm County and Karolinska Institutet, Stockholm, Sweden; Public Health Agency of Sweden, Stockhold, Sweden; Swedish National Food Agency, Uppsala, Sweden; Public Health Agency of Sweden, Stockholm, Sweden; Public Health Agency of Sweden, Stockholm, Sweden; European Programme for Intervention Epidemiology Training (EPIET), European Centre for Disease Prevention and Control (ECDC), Stockholm, Sweden; Public Health Agency of Sweden, Stockholm, Sweden

## Abstract

In June-July 2013, six counties notified the Swedish Institute for Communicable Disease Control of enterohaemorrhagic E.coli (EHEC) infections among attendees at a hotel in Dalarna, Sweden. An outbreak control team investigated to identify the source and implement control measures. We included individuals who attended the hotel between June 19th-25th in a cohort. We asked them about animal contact, swimming, and consumption of food items during this time using a questionnaire. A confirmed case was an EHEC O157:H7 outbreak strain positive individual who developed abdominal pain or diarrhoea between June 20th-July 2nd. We described the outbreak in time, place and person, calculated risk ratios (RR) and 95% confidence intervals (CI). We investigated the kitchen, tested and traced back implicated food items. 172 individuals responded. We identified 19 confirmed cases (Median age: 17 years, 64% female) with symptom onset between June 22nd-27th. Eating green salad on June 20th was associated with illness (RR:3.7;CI:1.3–11). The kitchen mixed green salads without records and destroyed leftovers immediately. Hence we could not conduct trace-back or obtain microbiological confirmation. Green salad contaminated before entering the kitchen was the likely outbreak source. We recommended early collaboration with food agencies and better restaurant records to facilitate future investigations.

## Introduction

Infections with enterohaemorrhagic Escherichia coli (EHEC), of which E.coli O157 is the most common strain^1^, occur worldwide^2^. The majority of cases are sporadic^2^. In 2012, among European Union countries, laboratory report rates of E.coli O157 infection ranged from <0.01/100,000 to 8.99/100,000^3^. Severity of EHEC infection ranges from being asymptomatic to abdominal cramps, bloody diarrhea, haemolytic uraemic syndrome (HUS) and death^1^. The median incubation period is 3-4 days^2^. A review of 90 confirmed outbreaks of EHEC infections from different parts of the world between 1982 and 2006 indicated that 54% of outbreaks were foodborne. Animal contact (8%) water (7%) and other environmental sources (2%) were less common^4^. In 29% of the reviewed outbreaks no source of infection was found^4^. In addition, 20% of outbreak cases on average resulted from secondary transmission^4^. Undercooked meat (especially beef), fermented meat products, milk and milk products, apple juice, grapes, coleslaw, lettuce, spinach, radishes, alfalfa sprouts, melons, and drinking or swimming in contaminated water have all been associated with EHEC infection outbreaks^5^.

In 1996, EHEC infection became a notifiable disease in Sweden. Half of the 350-500 annually reported cases are domestically acquired^6^. In 2005, a lettuce-associated outbreak of EHEC infection affected 135 people in Southwestern Sweden. Contaminated irrigation water was the likely source of infection^7^. Between 2010 and 2012, the reported rate of domestic cases increased and in 2012, 2.5 domestic EHEC infections per 100,000 population were reported^6^. E.coli O157 was the most common serogroup^6^.

On June 27^th^ 2013, the office for communicable disease control (SME) in Dalarna notified the Swedish Institute for Communicable Disease Control (SMI) of an EHEC infection in an individual who ate at hotel X in Rättvik, Dalarna County, Sweden. Hotel X welcomes private guests as well as large youth groups on a regular basis. On June 28^th^, Dalarna SME reported three additional individuals with EHEC infection who ate at the same hotel. On July 1^st^, Stockholm SME reported four individuals with EHEC infection who were part of a group that stayed at hotel X. Dalarna SME asked SMI to coordinate a national outbreak investigation. On July 5^th^, SMI posted information about the outbreak on its website. An outbreak control team investigated to estimate the magnitude of the outbreak, identify the source of infection and initiate control and preventive measures.

## Methods

The outbreak control team included SMEs from the six affected counties, Rättvik municipality environmental team, the National Food Agency (SLV), the National Board of Health and Welfare and members of the SMI epidemiology and food and waterborne disease units. The investigation was exempt from ethical committee review as it constituted an outbreak investigation response.

**Descriptive epidemiology**

To constitute a cohort, we reviewed hotel and restaurant bookings and listed individuals who attended hotel X between June 19th and June 25^th^, including staff. Each cohort participant received an email explaining about the outbreak, why we were asking them questions about their stay at the hotel, and explicitely stating replying to the questionnaire was voluntary. We included all individuals who consented and who attended the hotel during this time, from the moment of attendance to the hotel. We identified cases among respondents by cross checking name and age with SMI’s notification database.

*Case definition*

We defined a possible **case as a hotel X cohort participant who developed abdominal pain, watery diarrhea or bloody diarrhea between June 20^th^ 2013 and July 2^nd^ 2013.

We defined a confirmed **case as a possible case with a laboratory-confirmed infection with EHEC O157:H7, Verotoxin 2 positive, Verotoxin 1 negative and E.coli attaching and effacing (eae) positive.

We defined an asymptomatic infection as a hotel X cohort participant with a laboratory-confirmed infection with EHEC O157:H7, Verotoxin 2 positive, Verotoxin 1 negative and eae positive and who was not a possible case.

We defined a secondary case as an individual who developed abdominal pain, watery diarrhea or bloody diarrhea after July 2^nd^ 2013 with a household member who was a possible, confirmed or asymptomatic case.

*Case finding*

We searched for cases among hotel X attendees, using telephone interviews and an online questionnaire. We reviewed EHEC infections notified to SMI during the outbreak period for any links with hotel X. We encouraged possible cases to see a general practitioner and leave stool specimens. Some counties asked relatives of cases to provide a stool specimen. Additionally, on July 3^rd^, the National Board of Health and Welfare used the European Early Warning and Response System (EWRS) to send a message to nine countries from which tourists had visited hotel X during the outbreak period, encouraging them to report any EHEC infections in these tourists to SMI.

*Data analysis*

We described the distribution of cases by time, place and demographic characteristics. We identified days and meals the cases had attended to formulate hypotheses.

**Analytical epidemiology**

*Data collection*

Using an online questionnaire, we collected information among cohort participants regarding age, sex, symptoms, travel history, symptoms among household members, contact with animals, swimming and consumption of food items served at breakfast, lunch or dinner according to menu served to individuals and groups. We excluded individuals with no internet access.

*Data analysis*

Among cohort participants, we first compared individuals who attended the hotel on a specific day with others in terms of attack rates (confirmed cases only) through the calculation of risk ratios (RR) and their 95% confidence intervals (95% CI), using Mantel-Haenszel methods. We progressively restricted the analysis to days we suspected contamination had taken place, comparing individuals with specific exposures of interest with others. We used stratification to adjust for multiple exposures.

**Microbiological investigations**

We confirmed positive EHEC results and further characterized strains. We identified the outbreak strain using real time PCR^8^ and Multiple-Locus Variable Number Tandem Repeat Analysis (MLVA)^9^ and compared the identified MLVA profile with SMI’s database of strains previously identified in clinical isolates, in addition to a limited number of food isolates. We asked the National Veterinary Institute (SVA) to check for the outbreak MLVA profile in their database of previously isolated EHEC strains from Swedish farms and cattle. We collected fecal specimens from all kitchen staff.

**Environmental investigations**

We collected information from kitchen staff to identify food handlers and the meals they prepared using a questionnaire. We identified food items with a potential role in the outbreak from the lists of food items delivered to the hotel between June 12^th^to 24^th^.

On June 28^th^Rättvik environmental health section collected specimens of food items that were in storage in hotel X (i.e., tomatoes, cucumber, green salad, salad mix and minced meat) and sent them to SLV for analysis. At time of specimen collection, the hotel had discarded leftovers from the outbreak period and had switched vegetable suppliers. In addition, approximately 100 L of tap water was ultra-filtered and analysed for presence of E.coli and EHEC O157:H7.

On July 15^th^, we visited hotel X to observe kitchen layout, food preparation and storage.

On July 21^st^SLV collected and analysed salad specimens and 50L samples of irrigation water from two local salad producers that supplied hotel X.

## Results

**Descriptive epidemiology**

We identified 19 confirmed and 9 possible cases. Of these 28 cases, 18 (64%) were female. The median age was 17 years (range 8-67). We also identified one secondary case and 10 individuals with asymptomatic EHEC infections. Out of 28 possible and confirmed cases, 10 (36%) were private guests, 11 (39%) were part of a group and 7 (25%) were employed at hotel X, but none as a food handler. Cases originated from six counties (Figure 1). No cases in other European Union countries were identified through EWRS. Of the 19 confirmed cases, 16 (84%) responded to the questionnaire and were included in the cohort.

Each of the six counties sent questionnaires to their residents, although not all kept a record of questionnaires sent. While the exact number of questionnaires sent was unclear, we estimated that 200-250 persons received the questionnaire. 172 individuals answered, of which 25 were confirmed or possible cases (overall attack rate= 14%). The median age of respondents was 25 years and 113 (65.7%) were female. Of the 16 confirmed cases who answered the questionnaire, 11 (69%) reported having diarrhea, 11(69%) abdominal pain and 6 (38%) bloody diarrhea. One case developed HUS.

**Distribution of confirmed cases per county, Sweden, June-July 2013:**
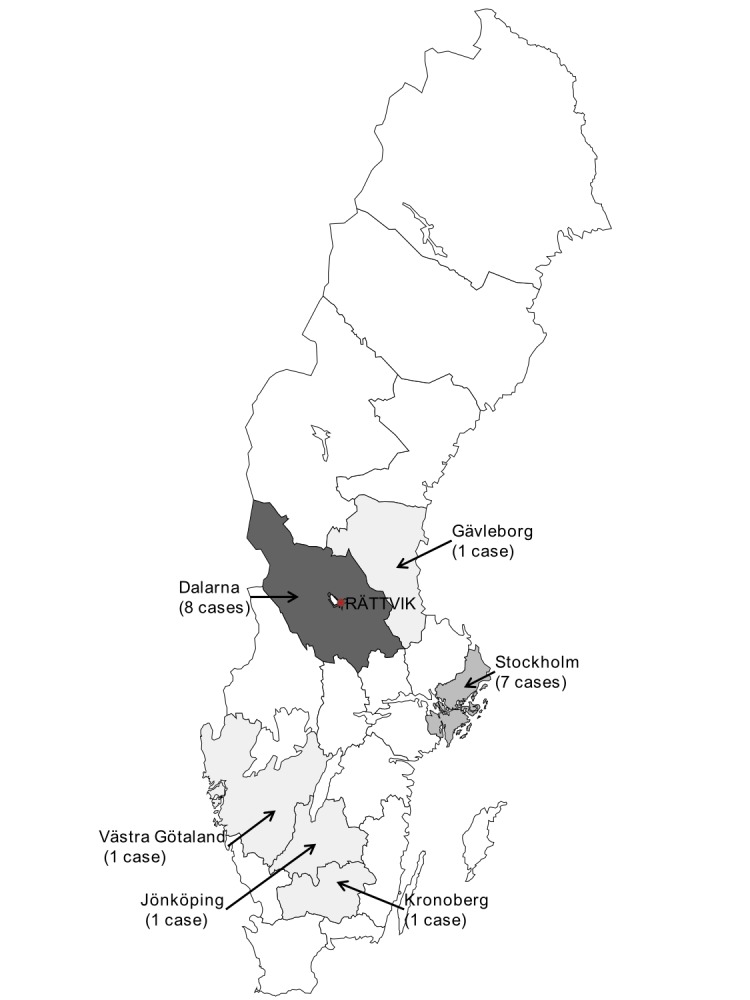

The earliest confirmed case occurred on June 22^nd^, the outbreak peaked on June 24^th^ and the latest confirmed case occurred on June 27^th^ (figure 2). The shape of the epidemic curve (figure 2) suggested a point source outbreak.

**Distribution of cases by date of onset, Dalarna, Sweden, June-July 2013:**
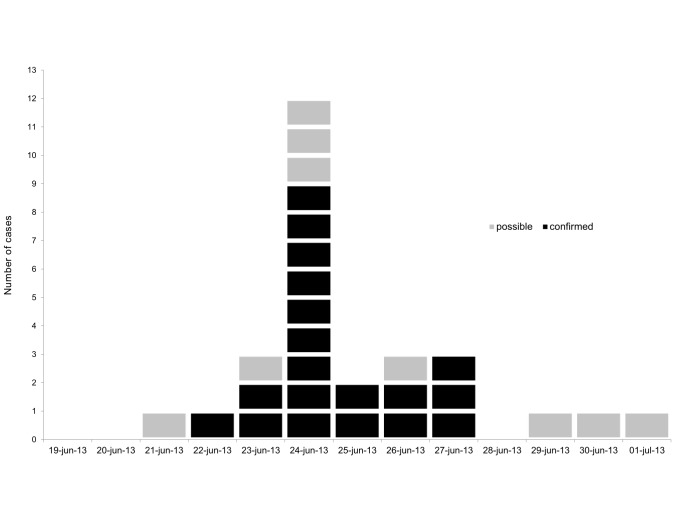

The confirmed cases attended between 1 and 16 meals during the outbreak period. Of the 16 confirmed cases who answered the questionnaire, 14 (88%) had eaten at least one meal on June 20^th ^(figure 3), of which 11 only had one meal (either breakfast, lunch or dinner). All cases had eaten at least one meal between June 19^th^ and June 21^st^. No single meal was attended by all cases.

**Proportion of confirmed cases attending each day at hotel X, Dalarna, Sweden, June 2013:**
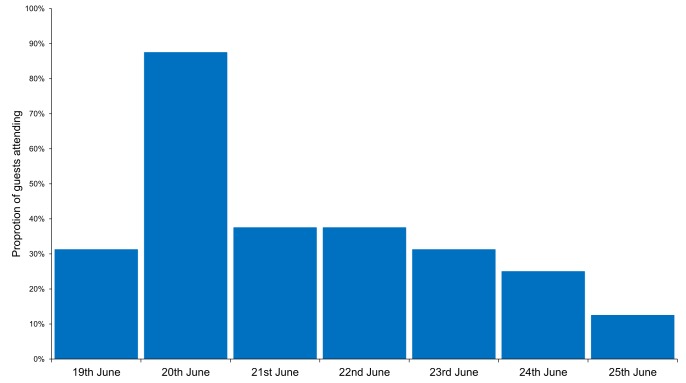

Regarding non food-related exposures, no cases reported contact with farm animals and 4/16 confirmed cases (25%) reported swimming in the lake adjacent to the hotel.

Since most cases attended a meal on June 20^th^, we hypothesized this was the most likely time of exposure. This date was also compatible with an outbreak peak on June 24^th^ given the median incubation period for EHEC infections.

**Analytical epidemiology**

Cohort members who attended Hotel X on June 20^th^ were more likely than others to be a confirmed case (RR 5.2, 95%CI 1.2-22, table I). 71% of confirmed cases were attributable to attending hotel X on this day. No specific meal was associated with being a confirmed case on that date (table 1). Swimming in the nearby lake was not associated with being a confirmed case (RR 0.52, 95% CI 0.17-1.5)

**Figure:**
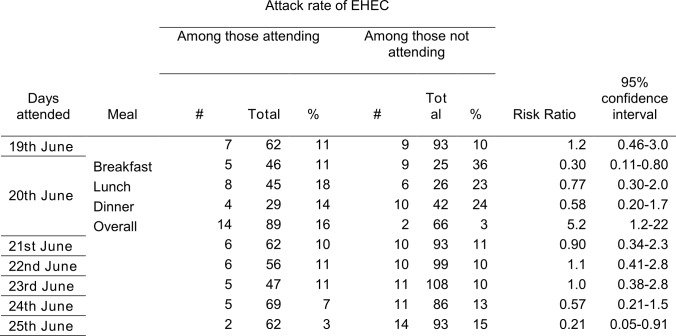
**Table 1. Attack rate of EHEC according to days attended at Hotel X and meals eaten, Dalarna, Sweden, June 2013**

On June 20^th^, Hotel X served a similar tex-mex buffet twice: once for lunch to private guests and once for dinner to groups. Individuals who ate chicken, beans, green salad or pineapple at the tex-mex buffet were more likely to be confirmed cases (RR 7.2, 3.0, 7.3 and 3.0 respectively, table 2). However, there was no association between eating chicken, beans or pineapple and being a confirmed case among those who ate green salad. Eating green salad at any meal on June 20^th^ was associated with being a confirmed case (RR 3.7, 95% CI 1.3-11, table 2). Of 23 individuals who ate green salad, 9 were confirmed cases (AR= 39 %) and 51% of confirmed cases attending hotel X on June 20^th^were attributable to eating green salad. Bean and lentil salad, pea salad and onion were also associated with being a confirmed case (Table 2) but their consumption only explained few cases. In addition, cases who ate these items also ate green salad. After adjusting for salad consumption, the risk ratios for all these items decreased (table 2) and when consumption of any of these items was treated as one exposure, individuals eating them were not at increased risk of being a case after adjusting for salad consumption (OR 5.3, 95%CI 0.72-39).

**Figure:**
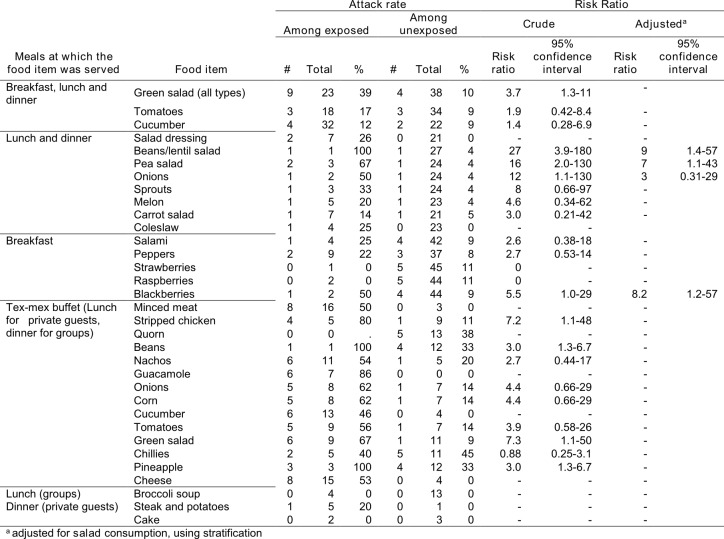
**Table 2. Attack rate of EHEC according to consumption of selected items on June 20th 2013 among guests attending these meals, Dalarna, Sweden**

**Laboratory investigations**

29 cases and asymptomatic individuals were laboratory confirmed with the EHEC outbreak profile. All shared the same MLVA profile: 6-7-13-4-6-6-6-4. This profile had not been previously seen in human, cattle, farms or food isolates in Sweden. Hence this was the first occurrence of this MLVA profile in Sweden. Routine surveillance identified two EHEC notifications in individuals with symptom onset on June 27^th^ and July 5^th^ with the same MLVA profile but who did not attend hotel X during the outbreak period. The rare MLVA profile suggested a link between these two individuals and the outbreak.

**Environmental investigations**

*Staff screening*

Of the 10 asymptomatic individuals, 5 were staff members. Of those, 3 handled food. Compared with others, individuals who ate meals prepared by any of the asymptomatic foodhandlers were not at higher risk of being a confirmed case (table 3).

**Figure:**
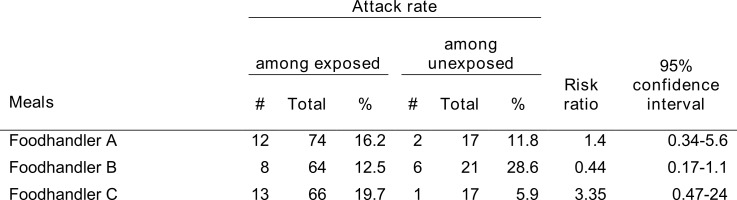
**Table 3. Attack rate of EHEC by consumption of meals prepared by specific food handlers among individuals who attended hotel X on the relevant days, Dalarna, Sweden, June 2013**

*Hotel inspection*

Youth groups and other guests ate in a separate dining room with separate menus. Staff could eat breakfast, lunch and dinner at the hotel. Hotel X had a single kitchen, with distinct areas for hot and cold food. There was a designated area to chop vegetables, but chopping board and knives were shared for all vegetables. Vegetables were often chopped at the same time for several meals, up to 48 hours in advance and stored in boxes in a refrigerated area. Different types of green salad were chopped and mixed together on the premises. Hotel X received whole and pre-chopped salads, but did not keep records of which types of salad were mixed and served on specific days. Food handlers wore gloves and washed salad prior to consumption. Salad was served in bowls, each with its own tongs. Unfinished salad bowls were stored in a refrigerated room and re-served at the next meal.

*Food trace-back and analysis*

Hotel X received different types of green salad from one supplier, the local branch of a nationwide vegetable supplier providing vegetables in the whole of Sweden from 6 regional delivery centers. This supplier in turn bought salad from one wholesaler as well as two local salad producers. The supplier pre-chopped salad at its head office in Stockholm. The hotel regularly received different types of green salads (iceberg, mixed leaves and Provence salad, both whole and pre-chopped), originating from France, Sweden and the Netherlands. Invoices documented all vegetables delivered to hotel X in terms of country of origin. In the absence of hotel records regarding the use of different green salads during specific meals, further trace back was not possible.

EHEC could not be detected in any of the food and water specimens from hotel X, or from salad specimens and irrigation water from the local salad producers.

**Analysis of cases unrelated to the cohort**

We interviewed the two individuals infected with EHEC with the same MLVA pattern but who did not attend hotel X.

The first person, with symptom onset on June 27^th^, lived more than 270 km away from hotel X. She had eaten lettuce and vegetables from her work canteen a few days before she fell ill although she could not remember the exact date or what type of salad. A different regional branch of the same vegetable wholesale company supplied the canteen, which was only one out of four places in the city where the company delivered vegetables.

The second person, with symptom onset on July 5^th^, lived more than 470 km away from hotel X area. We could not identify any potential link between this case and the hotel X outbreak.

## Discussion

Eating green salad on June 20^th^ 2013 was associated with being a confirmed case. A number of elements suggest that green salad was the most likely vehicle for this outbreak. First, eating green salad remained associated with being a confirmed case after adjustment for other exposures. Second, a high proportion of cases could be explained by eating salad and a high proportion of those who ate salad were confirmed cases. Third, green salad is a biologically plausible vehicle, having been identified as a vehicle for EHEC in at least seven outbreaks. between 2005 and 2013^,^ 7 ^,^ 10 ^,^ 11 ^,^ 12 ^,^ 13 ^,^ 14 ^,^ 15 ^,^ 16. Salads were prepared in advance and served from the same container over several meals around June 20^th^, and salad leftovers were re-served the following meal, which could explain a prolonged exposure over several meals/days. Hotel staff who were cases had dates of onset in the same time period as hotel guests. Hence they were likely infected at the same time from the same source.

Eating pea salad, bean/lentil salad or onions did not increase the risk of being a case after taking salad consumption into account. Additionally, we could not identify EHEC outbreaks linked to these food items. It is possible however that some of these items were contaminated in the kitchen when salads were prepared, since all cold salads were prepared in the same area, using the same utensils.

The vegetable supplier specified the origin of all vegetables supplied to hotel X, complying with EU regulation 178/2002 which requires the ability to trace and follow a food, feed, food-producing animal or substance intended to be, or expected to be incorporated into a food or feed, through all stages of production, processing and distribution^17^. However, in the EU, there is no statutory requirements for restaurants to keep a record of which salad was used in which meal. As such, we could not identify the salads used in each meal.

The salad distributor for hotel X was a nationwide chain that sourced salads locally and from abroad. An imported contaminated product distributed through a national distribution chain would have likely caused a higher number of sporadic cases across Sweden. Only two sporadic cases were however identified. Therefore, salad producers local to hotel X were the more likely source since there were only two cases out of the hotel X area. However, we could not link any specific salad producer to the outbreak.

Routine typing of all EHEC specimens has greatly facilitated the identification of the outbreak strain and of cases linked to the outbreak, highlighting the benefits of close collaboration between epidemiology and microbiology during outbreak investigations. In Sweden, foodborne outbreaks are routinely discussed via a central outbreak group bringing together epidemiology, microbiology and food safety services, facilitating such collaborations during outbreaks.

A salad batch brought contaminated to the hotel is the most likely outbreak source. The lack of food leftovers and the lack of record of which salad products were used in which meals prevented the identification of a more specific source. Trace-back in food produce associated outbreaks is a recurrent challenge. In 2012, in the EU, 6.3% of 5363 outbreaks investigated had the same causative agent identified in the food vehicle or food chain and in human cases^3^ . For fresh produce, microbiological confirmation is especially challenging given the short shelf life and rapid distribution and consumption, leading to loss of trace-back evidence^18^ . In addition, locally available produce may be globally sourced, and available in many other locations through wide distribution from one production area, complicating trace-back investigations^18^ . Successful trace-back requires an immediate alert and strict record keeping for specific food ingredients. In Sweden, these needs have not yet been translated into policy change.

On June 28^th^, environmental health services ordered the closure of hotel X. On July 5^th^, Hotel X reopened after disinfecting the kitchen and voluntarily changing meat and vegetables suppliers. On July 24^th^, after no new EHEC notifications with the same MLVA profile, SMI declared the outbreak over^19^. On the basis of our conclusions we recommend that (1) hotels and restaurants voluntarily keep a record of specific ingredients used in salad mixes and when they are served; (2) when suspecting food poisoning or an outbreak, hotels and restaurants immediately contact the food safety authorities before taking any measures such a disposing of food leftovers; In the absence of a regulatory framework, authorities at the local and central level could encourage catering businesses, hotels and restaurants to voluntarily implement these recommendations.

## References

[1] Pennington H. Escherichia coli O157. Lancet, 2010. 376(9750): 1428-35. 10.1016/S0140-6736(10)60963-4 20971366

[2] Heymann DL. Control of Communicable Diseases Manual, ed. A.P.H. Association. 2008, Washington: APHA Press.

[3] European Food safety Authority. The community summary report on trends and sources of zoonoses, zoonotic agents and food-borne outbreaks in the European Union in 2011. EFSA Journal, 2014. 12(2): 3547.

[4] Snedeker KG, Shaw DJ, Locking ME, Prescott RJ. Primary and secondary cases in Escherichia coli O157 outbreaks: a statistical analysis. BMC Infect Dis. 2009. 9: 144. 10.1186/1471-2334-9-144 PMC2741466 19715594

[5] Rangel JM, Sparling PH, Crowe C, Griffin PM, Swerdlow DL. Epidemiology of Escherichia coli O157:H7 outbreaks, United States, 1982-2002. Emerg Infect Dis, 2005. 11(4): 603-9. 10.3201/eid1104.040739 PMC3320345 15829201

[6] National Veterinary Institute. Surveillance of infectious diseases in animals and humans in Sweden 2012. 2012 [cited 2013 August 9 2013]. Available from: http://www.sva.se/upload/Redesign2011/Pdf/Om_SVA/publikationer/Surveillance2012.pdf.

[7] Söderström A, Osterbeg P, Lindqvist A, Jönsson B, Lindberg A, Blide ulander S et al. A large Escherichia coli O157 outbreak in Sweden associated with locally produced lettuce. Foodborne Pathog Dis, 2008. 5(3): 339-49. 10.1089/fpd.2007.0065 18767979

[8] Perelle S, Dilasser F, Grout J, Fach P. Detection by 5'-nuclease PCR of Shiga-toxin producing Escherichia coli O26, O55, O91, O103, O111, O113, O145 and O157:H7, associated with the world's most frequent clinical cases. Mol Cell Probes, 2004. 18(3): 185-92. 10.1016/j.mcp.2003.12.004 15135453

[9] Larsson JT, Torpdahl M, Petersen RF, Sorensen G, Lindstedt BA, Nielsen EM. Development of a new nomenclature for Salmonella typhimurium multilocus variable number of tandem repeats analysis (MLVA). Euro Surveill, 2009. 14(15). 19371515

[10] Taylor EV, Nguyen TA, Machesky KD, Koch E, Sotir MJ, Bohm SR et al. Multistate outbreak of Escherichia coli O145 infections associated with romaine lettuce consumption, 2010. J Food Prot, 2013. 76(6): 939-44. 10.4315/0362-028X.JFP-12-503 23726187

[11] Slayton RB, Turabelidze G, Bennett SD, Schwensohn CA, Yaffee AQ, Khan F, et al. Outbreak of Shiga toxin-producing Escherichia coli (STEC) O157:H7 associated with romaine lettuce consumption, 2011. PLoS One, 2013. 8(2): e55300. 10.1371/journal.pone.0055300 PMC3563629 23390525

[12] Sodha SV, Lynch M, Wannemuehler K, Leeper M, Malavet M, Schaffzin J et al. Multistate outbreak of Escherichia coli O157:H7 infections associated with a national fast-food chain, 2006: a study incorporating epidemiological and food source traceback results. Epidemiol Infect, 2011. 139(2): 309-16. 10.1017/S0950268810000920 20429971

[13] Ethelberg S, Lisby M, Bottiger B, Schultz AC, Villif A, Jensen T, et al. Outbreaks of gastroenteritis linked to lettuce, Denmark, January 2010. Euro Surveillance. 2010. 15(6). 20158982

[14] Friesema I, Sigmundsdottir G, van der Zwaluw K, Heuvelink A, Schimmer B, de Jager C et al. An international outbreak of Shiga toxin-producing Escherichia coli O157 infection due to lettuce, September-October 2007. Euro Surveillance. 2008. 13(50). 10.2807/ese.13.50.19065-en 19087865

[15] Uhlich GA, Sinclair JR, Warren NG, Chmielecki WA, Fratamico P, et al. Characterization of Shiga toxin-producing Escherichia coli isolates associated with two multistate food-borne outbreaks that occurred in 2006. Appl Environ Microbiol, 2008. 74(4): 1268-72. 10.1128/AEM.01618-07 PMC2258581 18083883

[16] Little CL, Gillespie IA. Prepared salads and public health. J Appl Microbiol, 2008. 105(6): 1729-43. 10.1111/j.1365-2672.2008.03801.x 18397258

[17] European Union. Regulation (EC) No 178/2002 of the European Parliament and of the Council. Official Journal of the European Communities 2002 [cited 2013 12th September 2013]; Available from: http://www.bfr.bund.de/cm/343/2002_178_en_efsa.pdf.

[18] Lynch MF, Tauxe RV, Hedberg CW. The growing burden of foodborne outbreaks due to contaminated fresh produce: risks and opportunities. Epidemiol Infect. 2009 Mar;137(3):307-15 10.1017/S0950268808001969 19200406

[19] Smittskyddsinstitutet. "Ehec-utbrott i Dalarna är över". 2013 [cited 2013 August 9 2013]; Available from: http://www.smittskyddsinstitutet.se/nyhetsarkiv/2013/myndigheter-utreder-utbrott-av-ehec-i-dalarna/.

